# Small molecules targeting endocytic uptake and recycling pathways

**DOI:** 10.3389/fcell.2023.1125801

**Published:** 2023-03-10

**Authors:** Giampaolo Placidi, Clara Mattu, Gianluca Ciardelli, Carlo C. Campa

**Affiliations:** ^1^ Italian Institute for Genomic Medicine, Candiolo, Italy; ^2^ Department of Mechanical and Aerospace Engineering, Politecnico di Torino, Turin, Italy; ^3^ Chemical-Physical Processes, National Research Council (CNR-IPCF), Pisa, Italy; ^4^ Candiolo Cancer Institute, FPO-IRCCS, Candiolo, Italy

**Keywords:** endocytosis, endocytic recycling, small molecules, inhibitors, mechanism of action

## Abstract

Over the past years a growing number of studies highlighted the pivotal role of intracellular trafficking in cell physiology. Among the distinct transport itineraries connecting the endocytic system, both internalization (endocytosis) and recycling (endocytic recycling) pathways were found fundamental to ensure cellular sensing, cell-to-cell communication, cellular division, and collective cell migration in tissue specific-contexts. Consistently, the dysregulation of endocytic trafficking pathways is correlated with several human diseases including both cancers and neurodegeneration. Aimed at suppress specific intracellular trafficking routes involved in disease onset and progression, huge efforts have been made to identify small molecule inhibitors with suitable pharmacological properties for *in vivo* administration. Here, we review most used drugs and recently discovered small molecules able to block endocytosis and endocytic recycling pathways. We characterize such pharmacological inhibitors by emphasizing their target specificity, molecular affinity, biological activity and efficacy in both *in vitro* and *in vivo* experimental models.

## Introduction

Endocytic membrane trafficking plays an essential role in delivering both solute molecules and membrane components (e.g., lipids and proteins) to various intracellular destinations (Doherty and McMahon, 2009; Haucke, 2015). Conceptually, endocytic trafficking routes are relatively simple, with the main pathways carrying either to degradation in lysosomes or to recycle back to the plasma membrane. However, genetic redundancy and pleiotropy profoundly impact on molecular organization of membrane trafficking, thus limiting identification of both specialized trafficking itineraries and pivotal protein interactions (Yarwood et al., 2020).

Endocytic membrane trafficking is a ubiquitous process in eucaryotic organisms. Non-etheless, not all cells respond to perturbation of membrane trafficking machinery in the same way (De Matteis and Luini, 2011; Yarwood et al., 2020; García-Cazorla et al., 2022). This is posited to depend on: 1) the abundance and the degree of functional redundancy of membrane trafficking components, and 2) the relevance of the transported cargo for cellular functions and tissue homeostasis. For instance, the nervous system is susceptible to disruption of endocytic genes involved in both endocytic recycling and autophagy. This is due to both the low proliferating activity and the elevated speed of neurotransmitters secretion/internalization that characterize neuronal cells (Schreij et al., 2016). Consequently, impairment in endocytic trafficking pathways significantly affect behaviour and function of the nervous tissue, often resulting in neurological disorders including frontotemporal dementia (FTD), Alzheimer and Parkinson diseases (Harold et al., 2009; DeJesus-Hernandez et al., 2011; Vilariño-Güell et al., 2011; Vilariño-Güell et al., 2014). Similarly, alteration in endocytic recycling significantly impact in cells with elevated secretory activity, such as pancreatic beta cells. In particular, mutations in AS160, an endocytic recycling regulator, impairs the translocation of the glucose transporter GLUT4, thus leading to defective glucose blood clearance and hence muscle insulin-resistance and type 2 diabetes (Karlsson et al., 2005; Mîinea et al., 2005; Moltke et al., 2014). Despite these considerations, the employment of membrane trafficking alterations to predict both type and status of a disease is far from being achieved.

The therapeutic targeting of membrane trafficking pathways might be used to increase both delivery and efficacy of currently employed therapeutics for cancer, respiratory disorders and, concomitantly, to provide novel strategies for the treatment of over 300 Mendelian genetic diseases (Chew et al., 2020; Kang et al., 2020; Riva et al., 2020; García-Cazorla et al., 2022). Unfortunately, the complex organization of the endomembrane system limits the identification of membrane trafficking inhibitors using phenotype-based screening strategies. In parallel, it remains still difficult to identify druggable molecular mechanisms pivotal for transport of specific molecular cargoes (De Matteis et al., 2013; Mishev et al., 2013).

This review provides an overview of main drugs controlling regulatory functions of endocytic proteins. This is particularly important in view of recent progress into endocytic trafficking field. To get insights into mechanisms characterizing novel transport itineraries that are not covered by this review, we remand readers to these excellent works (Redpath et al., 2020; Renard and Boucrot, 2021; Sigismund et al., 2021; Gilleron and Zeigerer, 2023).

## Targets for endocytic recycling

The internalization and their transport back to the plasma membrane of both solute molecules and membrane components (e.g., lipids and proteins) is carryout by both the endocytic and the recycling membrane trafficking machineries, respectively. Every transport itinerary requires the budding, scission and transport of vesicles from the donor compartment and their subsequent targeting, tethering, docking and fusion to the acceptor membrane. Notably, these trafficking steps are controlled by protein subsets that, according to their roles during the transport process, can be organized into specific functional modules. Below, we provide description of building blocks used by both endocytic and recycling pathways. This list encompass both adaptors, coats, shaping, fission, small GTPase, kinase, tethering and fusion proteins involved in endocytic recycling (Liberali et al., 2008).

### Adaptor module

Both AP180, *β*-arrestin, NUMB, HIP1, DAB2, ARH, EPSIN1, EPS-15 proteins and components of the adaptor complexes AP1, AP2, AP3 play a central role in cargo recognition and membrane-coat complex assembly during endocytic recycling (Reider and Wendland, 2011). These adaptors are composed by different protein domains which mediate interactions with membrane lipids (e.g., phosphatidylinositol 4,5-bisphosphate), coats (e.g., clathrin) and cargo proteins (e.g., G-protein coupled receptors, tyrosine kinase receptors). Notably, adaptor recruitment to cargoes can be controlled by post-translational modifications (e.g., phosphorylation), thus ensuring internalization of signalling receptors following agonist stimulation.

### Coat module

This functional module includes clathrin (both light and heavy chains), caveolin and flotillin, each of which is recruited to both plasma membrane and endosomes by several endocytic adaptor subsets. Specific protein-binding motifs enable both clathrin polymerization and caveolin/flotillin oligomerization into higher-order structures (i.e., basket, disk-shaped or tetramers, respectively), thus helping the budding of vesicles from donor membranes (Van Jaarsveld et al., 1981; Chaudhary et al., 2014; Kononenko et al., 2014; Watanabe et al., 2014; Shvets et al., 2015; Han et al., 2020; Porta et al., 2022; Singh et al., 2022).

### Shaping module

Bin/amphiphysin/Rvs (BAR) domain-containing proteins play a prominent role in membrane remodelling in response to protein surface density, membrane tension, or membrane shape alterations. Arfaptin, Amphiphysin, Sortin Nexins, Endophilin, FCHo proteins are the most pervasive membrane-shaping regulators controlling the invagination of both cell surface and endocytic membranes. In addition to the BAR domain, such membrane remodelling proteins can encode a small GTPase-regulatory region. For instance, ASAP and ACAP proteins display an Arf-GAP domain which allow functional coupling between BAR-mediated membrane sculpting and actin polymerization, specifically. Lastly, while the mentioned proteins control endocytic vesicle formation on both endosome and plasma membrane, a different BAR domain-containing proteins family (e.g., MIM, IRTKS, IRSp53) evolved to direct the generation of plasma membrane protrusion (Simunovic et al., 2015; Renard et al., 2018; Simunovic et al., 2019).

### Fission module

The membrane fission module relies on mechanoenzymatic machineries that bind phosphatidylinositol-enriched membranes to favour membrane constriction through a GTP-dependent mechanism. Dynamin proteins (e.g., DNM1, DNM2, DNM3) are GTPase enzymes that hydrolyse GTP to prompt vesicle fissions in endocytic membrane-bound compartments. DNM1 and DNM3 are enriched in brain and they both mediate retrieval of synaptic vesicle membranes, an event that occurs in concomitance with exocytic fusion and cargo recycling to the cell surface. In contrast, DNM2 is ubiquitously expressed and mediates endocytic uptake (Ferguson and De Camilli, 2012; Wu et al., 2014; Lee et al., 2016; Renard et al., 2018; Jimah and Hinshaw, 2019; Imoto et al., 2022).

### Rab GTPase modules

The RabGTPase module comprises over 60 distinct genes in the human genome. Rab GTPases are molecular switches that, by cycling between an active and inactive state, they recruit, on membranes, specific molecular effectors. Through their effectors, Rab GTPases regulate vesicle formation, vesicle movement along actin and tubulin networks, and membrane fusion. Notably, specific Rab GTPases subsets are localized to different membrane-bound compartments and hence they act as signpost to recognize transport itineraries in mammalian cells. In this context, RAB4 family (A, B, C), RAB5 family (A, B, C), RAB13, RAB 20, RAB21, RAB22 family (A, B), and RAB23 are restricted to early endosomes, while the recycling compartment stained positive for RAB3 family (A, B, C, D), RAB8 family (A, B), RAB10, RAB11 family (A, B, C/RAB25), RAB14, RAB15, RAB17, RAB35 (Wandinger-Ness and Zerial, 2014).

### Kinase module

This module comprises both protein and lipid kinases controlling phosphorylation, and hence activation, of either protein or lipid-signalling, in response to cell’s environmental changes. As an example, the phosphorylation of Rab GDP dissociation inhibitor (GDI) by p38 (MAPK11, -13, -14), a stress-activated protein kinase, causes Rab5 cytosolic sequestration and endocytosis blockage. As a further example, dynamins are substrates for both receptor-activated kinases (e.g., SRC, casein kinase, GSK3) and cell cycle regulated kinases (e.g., CDK5, DYRK1), thus providing inducible control in vesicle fission. In parallel, the lipid kinases-mediated generation of signalling lipids promotes maturation of endocytic membranes through the recruitment of both membrane-associated and cytosolic proteins. In this context, phosphatidylinositol 4-phosphate 5-kinase (PI4P5K) and phosphatidylinositol 5-phosphate 4-kinase (PI5P4K) enzymes generates phosphatidylinositol 4,5 biphosphate [PtdIns(4,5)P2], the major signalling lipid involved in endocytosis and essential for plasma membrane association of AP2 complex (Cavalli et al., 2001; Clayton et al., 2010; Liberali et al., 2014; Wallroth and Haucke, 2018; Perez Verdaguer et al., 2021).

### Tethering module

Two distinct molecular classes control long-distance vesicle recognition to acceptors compartment: coiled-coil proteins and multi-subunit complexes. EEA1 and Rabphilin-3A are prototypical coiled-coil proteins involved in fusion of endocytic vesicles to early endosomes and plasma membrane, respectively. In parallel, multi-subunit complexes such as CORVET promotes tethering and fusion of Rab5/Vps21-positive membranes, while Exocyst, EARP, FERARI control the fusion of endocytic vesicles to plasma membrane (Peplowska et al., 2007; Balderhaar et al., 2013; Schindler et al., 2015; Ahmed et al., 2018; Solinger et al., 2022).

### Fusion module

The fusion module comprises SNARE (soluble N-ethylmaleimide-sensitive factor attachment protein receptor)-motif containing proteins that mediate vesicles fusion to acceptor membranes. Previously named as v- and t- SNARE, due to their localization to either vesicle or target membranes, while currently classified into Qa-, Qb, Qc- and R-SNARE based on their sequence features, SNARE proteins bind to each other to form a parallel four-helix bundle. This helix structure, bridges and ultimately fuses vesicle membranes. The endocytic fusion machinery requires Syntaxin (Stx), VAMP and SNAP-25 protein homologues such as: Stx13, Stx6, Stx2, Stx4, Vti1a, VAMP4, and VAMP7. Targeting specificity is determined by the combinatorial assembly of the over 35 different SNAREs. Nonetheless, recent reports indicate that some SNAREs can functionally substitute for each other if they belong to the same subfamily (Jahn and Scheller, 2006; Koike and Jahn, 2017; Koike and Jahn, 2019).

## Small molecules targeting the endocytic machinery

Internalization and delivery of both protein and lipids to intracellular membrane-bound compartments are fundamental for cell physiology (Holter, 1959; Harding et al., 1983; Sigismund et al., 2012; Shafaq-Zadah et al., 2020). Such endocytic itineraries are classified based on the molecular machinery employed, principally either clathrin or dynamin -based. To date, five major endocytic trafficking routes are identified: 1) clathrin-mediated endocytosis (CME; clathrin and dynamin dependent), 2) caveolae-mediated endocytosis (CavMe, clathrin independent and dynamin dependent), 3) fast endophilin-mediated endocytosis (FEME, a clathrin-independent but dynamin-dependent pathway), 4) clathrin-independent carrier (CLIC)/glycosylphosphatidylinositol-anchored protein enriched early endocytic compartment (GEEC) endocytosis (clathrin and dynamin independent), 5) macropinocytosis and 6) phagocytosis (Cardoso et al., 2021). Due to the fundamental role of both coat and pinch-off proteins, most efforts have been directed towards the discovery of small molecules targeting both clathrin and dynamin pathways. However, beside these rationally-designed compounds, many drugs, with unknown target specificity and able to interfere with trafficking pathways, are still employed in membrane trafficking filed. For instance, Genistein (4′,5,7-trihydroxyisoflavone), a molecule that belong to tyrosine kinase inhibitors, hamper caveolae-mediated endocytosis and partially clathrin-mediated endocytosis by an unknown mechanism (DeLouise, 2012). In addition, Methyl-b-cyclodextrin (MbCD), a cyclic heptasaccharid, depletes cholesterol from cell membrane causing lipid-rafts/caveolin depletion and the subsequent perturbation of both clathrin- and caveolae-mediated pathways (Vercauteren et al., 2010).

In the section below, we describe main endocytic inhibitors characterized by elevated target specificity. In this summary, we not include either molecules or treatment, such as potassium depletion or sucrose treatment (Rennick et al., 2021), responsible for internalization defects by unknown mechanisms of function (Supplementary Figure S1; Table 1).

**TABLE 1:** Small molecules inhibitors targeting endocytic molecules.

Molecule	Target	Mechanism of action	Effect on endocytic pathway	Effect on membrane	Use in cells and animals	Clinical trial	Reference
*Pitstop2*	Clathrin heavy chain	Inhibits interaction between N-terminal domain of Clathrin and its accessory proteins (Amphysin, AP180, Synaptojanin, OCRL)	Inhibits clathrin- mediated endocytosis and partially clathrin-independent endocytosis	Blocks maturation of clathrin-coated vesicles	10 μM (§)	None	von Kleist et al. (2011), Paksoy et al. (2022), Dutta et al. (2012), Willox et al. (2014), Liashkovich et al. (2015)
	12 μM (*)				Delivered by stereotaxic injections in rats at 120 µM for2 µl (#)		
*Endosidin 9–17*	Clathrin heavy chain 1	Inhibits clathrin-coated pits formation presumably by interacting with AP2 complex	Inhibits clathrin-mediated endocytosis	none	17.2 μM (§)	None	Dejonghe et al. (2016), Dejonghe et al. (2019)
*Barbadin*	β-arrestin/β2-adaptin interaction	Inhibits *β*-arrestin/β2-adaptin interaction	Inhibits agonist-induced GPCR endocytosis such as β2-adrenergic (β2AR), V2-vasopressin (V2R) and angiotensin-II type-1 (AT1R) receptors	Reduces plasma membrane PI(4,5)P2 and membrane invagination	15–19 μM (§) 0.3 mg/kg (#)	None	Beautrait et al. (2017), He et al. (2021), Jung et al. (2021)
*Dyngo-4a*	Dynamin I, II	Non-competitive dynamin inhibitor. Binds allosteric sites on the G domain. Inhibits helical dynamin oligomerization states	Blocks dynamin-dependent endocytosis, including CME and activity dependent bulk endocytosis	Mithocondrial stress	30 µM (§) 30 mg/kg (#)	None	McCluskey et al. (2013), von Beek et al. (2021)
	0.38 and 2.3 μM respectively, (*)						
*Dynasore*	Dynamin I, II	Non-competitive dynamin inhibitor. Inhibits dynamin oligomerization	Blocks dynamin-dependent endocytosis, including CME.	Reduces plasma membrane cholesterol. Causes membrane tubulation	80 µM (§) 10 mg/kg (#)	None	Macia et al. (2006), Kirchhausen et al. (2008), McCluskey et al. (2013), Preta et al. (2015), Zhong et al. (2019)
	15 μM (*)						
*Dynole34-2*	Dynamin I, II	Non-competitive dynamin inhibitor. Binds allosteric sites on the G domain	Blocks dynamin-dependent endocytosis, including CME and receptor mediated endocytosis	Induces membrane protrusion	20 µM (§) 30 mg/kg (#)	None	Chircop et al. (2011), Harper et al. (2011), McCluskey et al. (2013), Basagiannis et al. (2021)
	1.4 and 41.1 μM, respectively (*)						
*Pthaladyn-23*	Dynamin I	GTP-competitive inhibitor	Inhibits clathrin-mediated endocytosis and synaptic vesicle endocytosis	Not reported	Not reported	None	Odell et al. (2010)
	17.4 μM (*)						
*Chlorpromazine*	Dynamin I, II	Lipid-competitive dynamin inhibitor. Blocks lipid-stimulated dynamin activity	Inhibits clathrin-mediated endocytosis CME of TfR, EGFR, and Notch receptors	Cause cell membrane deformation	10–40 µM (§) 7.5 mg/kg (#)	FDA approved for psychotic diseases	Shin et al. (2013), Daniel et al. (2015), Kazama et al. (2015), Oliva et al. (2017)
	6.8 µM (*)						
*MiTMAB*	Dynamin I, II	Lipid-competitive dynamin inhibitor. Blocks lipid-stimulated dynamin activity	Inhibits clathrin-mediated endocytosis CME	Not reported	50 µM (§) 10 mg/kg (#)	None	von Beek et al. (2021), Joshi et al. (2010), Hill et al. (2004), Quan et al. (2007), Joshi et al. (2011)
	3.1 and 8.4 µM , respectively (*)						
*Pitcoin 3*	PI3KC2α	ATP-competitive inhibitor. Block synthesis of PI(3,4)P2, PI(3)P lipids	Inhibits clathrin-mediated endocytosis	Affects Platelets morphology	20 µM (§)	None	Lo et al. (2022)
	4.6 µM (*)						
*CT99021 (CHIRs)*	Glycogen synthase kinase 3-B	ATP competitive Inhibitor. Blocks Dynamin I phosphorylation	Inhibits activity-dependent bulk endocytosis (ADBE) but not clathrin-mediated endocytosis	None	5 µM (§) 25 mg/kg (#)	None	Heap and Cowan (1991), Clayton et al. (2010)
	34 nM (*)						
*SB203580 (Adezmapimod)*	P38 MAPK	ATP-competitive inhibitor. Blocks p38-mediated activation of RAB-GDI	Delayed endocytosis. Inhibits endosome to Golgi trafficking	Cell shape alteration	10 µM (§) 25 mg/kg (#)	None	Cavalli et al. (2001), Wälchli et al. (2008)
	500 nM (*)						

(*): IC_50_ in recombinant protein assay.

(§): in mammalian cells.

(#): in rodents.

### Clathrin heavy chain inhibitors

Clathrin heavy chain (CHC) is a self-assembling protein that coats transport vesicles during their endocytic sorting. The assembly of clathrin coat requires interaction between CHC and clathrin-associated sorting proteins (CLASPs) through the CHC terminal domain (TD) (Schmid and McMahon, 2007; Ranjan et al., 2017). CLASPs comprise many endocytic proteins such as the AP2 complex, AP180, CALM, Eps15, Amphiphysin involved in internalization of many growth factor receptors (Lemmon and Traub, 2012). CLASPs localization guides clathrin basket formation at distinct cellular compartments, allowing directionality in membrane trafficking flow. As an example, clathrin-coated vesicles (CCVs), programmed for being delivered from cell surface and internal organelles, are assembled on both endosomes and plasma membrane by specific adaptor proteins such as AP1B1 (designated β1) and AP2B2 (β2), respectively. Based on the important role played by CHC and CLASPs binding for CCVs assembly, huge efforts were made to identify compounds able to block the interaction between CHC’s TD and CLASPs, of which Pitstop2 and Endosidin9-17 are the most noticeable.

Pitstop2 associates with the clathrin TD and obstructs binding of accessory proteins involved in both maturation and disassembly of CCP such as Amphysin, AP180, Synaptojanin, OCRL (von Kleist et al., 2011). In cells, Pitstop2 blocks clathrin-mediated endocytosis and generates enlarged endosomes when used in rodents and specifically at the calyx of Held, a synapse optimized for high frequency synaptic transmission in the auditory brainstem (Paksoy et al., 2022). Nonetheless, to date several clathrin-independent effects were reported for Pitstop2 (Dutta et al., 2012).

A similar mechanism of function is employed by Endosidin9 (ES9), a small molecule that induces, after short exposure (∼30 min), both clathrin and AP2 mislocalization in human, plants and fruit-fly cells (Dejonghe et al., 2016). Notably, ES9-17, a ES9 more potent derivative, was demonstrated to block endocytosis in *Arabidopsis Thaliana*, a model system resistant to Pitstop2 inhibition due to an aminoacidic substitution (residue 80) in plant’s clathrin heavy chain isoform.

### Caveolin inhibitors

Caveolins are integral membrane proteins that play an important structural role in caveolae-mediated endocytosis, an endocytic process mediating the internalization of Cholera toxin, Ebola, Hepatitis B, Japanese encephalitis, human coronaviruses -229E and -OC43 (Xing et al., 2020; Parton et al., 2021). Three distinct caveolin isoforms were identified (Cav1, Cav2, Cav3) and found differentially expressed in human tissues. In particular, Cav1 is enriched in brain, skeletal muscle, liver, stomach, lung, kidney and heart. Cav2 is predominantly expressed in endothelial cells, smooth muscle cells, and fibroblasts, while Cav three is found in muscular tissue (Scherer et al., 1997; McMahon et al., 2009). Caveolins are composed by a transmembrane region flanked by two (N-, C-) terminal domains, each of them is exposed to cell’s cytoplasmic side and involved in caveolin post-transaltional regulation. The transmembrane portion comprises an oligomerization domain and a scaffolding domain which control, respectively, caveolin oligomerization and cholesterol binding (Parton and del Pozo, 2013; Porta et al., 2022). Both these protein-protein and protein-lipid interactions are required for caveolin-mediated caveolae formation. The oligomerization domain mediates the aggregation of caveolin in 14–16-unit oligomers (Porta et al., 2022), while the scaffolding portion allows both membrane insertion into cholesterol-enriched membrane regions and interaction with cholesterol-binding proteins (Krishna and Sengupta, 2019).

Currently, two distinct strategies are available to block caveolin activity during endocytosis: cholesterol inhibition and caveolin-oligomer disruption. The first employs Methyl-b-cyclodextrin, FilipinIII and Nyastatin to deplete cholesterol and ergosterol from cellular membranes, while the second uses WL47, a synthetic peptide, to reduce *in-vitro* caveolin oligomers assembly (Plummer and Manchester, 2013; Gilliam et al., 2016).

### CLASPs inhibitors

The interaction between clathrin-associated sorting proteins (CLASPs) and clathrin guides the internalization of cell surface proteins. Both *β*-arrestins and AP2 complex bind clathrin and both are required to induce internalization of cell surface receptors. Specifically, *β*-arrestins are activated downstream G-protein coupled receptors (GPCRs). Following sustained agonist stimulation, *β*-arrestin are recruited at the plasma membrane by phosphorylated-activated GPCRs. This event promotes functionally uncoupling of the activated receptors from their heterotrimeric G proteins (Lohse et al., 1990). Subsequently, the association of *β*-arrestins to AP2 induces GPCR internalization and trafficking towards endosomes for their degradation/recycling.

Barbadin is a small molecule that targets the contact interface between *β*-arrestin and AP2. Barbadin blocks GPCR internalization without affecting trafficking of Transferrin receptor (TfR), a non-GPCR that interacts directly with AP2 in a *β*-arrestin independent manner (Jing et al., 1990). In a preclinical experiment, Barbadin was demonstrated to potentiate the effects of Lorcaserin, a serotonin 2C receptor (5-HT2CR) selective agonist. Barbadin treatment inhibits 5-HT2CR internalization after lorcaserin stimulation, maintaining proper proopiomelanocortin (POMC) neuron responses to serotonin-agonist challenge *in vivo*, ultimately leading to appetite reduction and weight gain in mouse models (He et al., 2021).

### Dynamin inhibitors

Dynamins are large GTPase proteins that mediate membrane fission and fusion during endocytosis, recycling, and organelle biogenesis (Jimah and Hinshaw, 2019). Three distinct dynamin isoforms were identified and found differentially expressed in human tissues. Dynamin I is mostly expressed in brain-related tissues, Dynamin II is ubiquitous, whereas Dynamin III is enriched in brain, lungs, and testis (Ferguson and De Camilli, 2012). All dynamin isoforms contain a GTPase (G) domain involved in GTP binding and hydrolyses, a pleckstrin-homology (PH) domain which recognizes PtdIns(4,5)P2, a membrane lipid, and a GTPase effector domain (GED) that allows oligomerization and stimulation of Dynamin GTPase activity (Jimah and Hinshaw, 2019). Three distinct classes of small molecule inhibitors were developed to differentially target either dynamin’s oligomerization, GTPase activity or lipid-stimulated GTPase activity (Table 1).

Dyngo-4A and Dynasore are small molecules that, by binding allosteric sites in the Dynamin G domain, block dynamin oligomerization into either ring-like or helical structures, thereby impeding cooperative GTP hydrolysis, membrane tubulation and hence clathrin-mediated endocytosis, as demonstrated by reduction of TfR uptake (Tuma and Collins, 1994; Hinshaw and Schmid, 1995; Macia et al., 2006; McCluskey et al., 2013). A different mechanism is adopted by Chlorpromazine, a phenothiazine-derived antipsychotic drug and MiTMAB. These compounds block lipid-stimulated Dynamin GTPase activity by competing with lipid binding (Daniel et al., 2015). In parallel, competitive inhibition of GTP nucleotide binding is used by Pthaladyn-23 (Odell et al., 2010).

One of the potential employments of dynamin inhibitors is the boost of antibody-dependent cellular cytotoxicity (ADCC) response for cancer treatment. By blocking internalization of cell surface antigens, dynamin inhibitors increase target receptor availability for antibody binding. Thus, retention of antigen-antibody complexes enhances recognition of cancer cells by natural killer (NK) cells. These findings were corroborated in preclinical experiments by using Dyngo-4a and prochloropyrazine, a less toxic chlorpromazine analogue. These dynamin inhibitors were found effective in combination with cetuximab, trastuzumab and avelumab, three distinct clinical-approved antibody-based treatment targeting EGFR, HER2 and PD-L1 receptors, respectively (Chew et al., 2020). Although impressive results were obtained by these compounds, dynamin inhibitors are not isoform-selective and hence not able to block specific trafficking pathways. Since dynamin isoforms act on distinct cellular districts (Gray et al., 2003), major concerns regarding their on-target toxicity during endocytic recycling are present.

### Kinase inhibitors

Kinases are enzymes that catalyse the transfer of phosphate groups to either proteins or lipids. As a result, kinases activity is rarely limited to endocytic pathways, often resulting in perturbation of several cellular processes (Roskoski, 2015). Nonetheless, the kinase-induced phosphorylation mechanism permits the endocytic machinery to react to changing cellular demands and hence ensuring appropriate cell’s response to a fluctuating environment (Roskoski, 2015).

CT99021 is an inhibitor of Glycogen synthase kinase (GSK3B), a serine-threonine kinase regulating the phosphorylation of more than 100 substrates including both cell proliferation and membrane trafficking pathway components (Chen et al., 2007; Beurel et al., 2015; Hermida et al., 2017; Zheng and Conner, 2018a). CT99021 blocks GSK3B-mediated phosphorylation of Dynamin I at Ser-774 resulting in activity-dependent bulk endocytosis (ADBE) defects (Clayton et al., 2010). Notably, CT99021-mediated GSK3B inhibition is currently used in preclinical models to investigate the potential of endocytosis blockade in depression treatment, spatial learning and memory amelioration (Smillie et al., 2013; Lee et al., 2021). In this context, synaptic injury and cognitive decline can be rescued by SB203850, a p38 MAP kinases (MAPK11-14) inhibitor, that delay Rab5-mediated endocytosis through RAB-GDI inactivation (Cavalli et al., 2001; Wälchli et al., 2008; Yu et al., 2018).

Differently to other protein kinases, lipid kinases catalyse the ATP-dependent transferase reaction on lipid membranes, thereby localizing signalling reactions on membrane surfaces (Feng and Yu, 2021; Yoshioka, 2021; Burke et al., 2022). Therefore, alteration of lipid kinases activity is rarely associated with either inhibition or mistargeting of specific trafficking routes (Ronan et al., 2014). Nonetheless, Pitcoin3, an ATP-competitive PI3KC2α inhibitor, was recently developed to block clathrin-mediated endocytosis. By reducing plasma membrane and endosomal phosphoinositide’s content (i.e., PtdIns(3,4)P2 and PtdIns(3)P) Pitcoin3 mimicks the effects of enzyme’s genetic loss (Posor et al., 2013; Campa et al., 2018; Yoshioka, 2021; Lo et al., 2022).

## Small molecules targeting the endocytic recycling machinery

Delivery of endocytosed plasma membrane components to various intracellular compartments is achieved by a multitude of trafficking pathways. Based on the final cargo’s destination, such intracellular trafficking routes are classified in the degradative, retrograde and recycling routes (Scott et al., 2014). In this section, we describe lysosomotropic agents (e.g., bafilomycin A1, NH4Cl, chloroquine, ionomycin, nigericin, monensin) and small molecules affecting endocytic recycling (Johnson et al., 1993; Scott and Gruenberg, 2011) (Supplementary Figure S2; Table 2).

**TABLE 2:** Small molecules inhibitors targeting specific recycling modules.

Molecule	Target	Mechanism of action	Effect on endocytic pathway	Effect on membrane	Use in cells and animals	Clinical trial	Reference
*Primaquine*	Unknown	Neutralizes the endosomal pH	Causes partial inhibition of recycling	Induce endosomal enlarged vacuoles	10 µM (§) 0.2 mg/kg (#)	Approved as antimalarial agent and for COVID-19 treatment	van Weert et al. (1995), van Weert et al. (2000), Bancone et al. (2016), Mishra et al. (2022)
*Monensin*	Unknown	Neutralizes the endosomal pH	Causes partial inhibition of recycling	Golgi aberrations and ER stress		None	Wang et al. (2018), Vanneste et al. (2019)
*Nigericin*	Unknown	Neutralizes the endosomal pH	Causes partial inhibition of recycling	Golgi aberration and ER stress		None	Podinovskaia et al. (2021)
*Bafilomycin A1*	V-ATPase 0.44 nM (*)	Disrupts the interactions between c-ring and a region of V0 V-ATPase subunit	Causes partial inhibition of recycling	ER fragmentation in small rounded membranes	100 nM (§) 1 mg/kg (#)	Not yet recruiting phase for COVID-19 treatment	Yoshimori et al. (1991), Yuan et al. (2015), Wang et al. (2021), Dive et al. (2022)
*Endosidin2*	EXO70A1	Inhibits protein-protein interaction between EXO70A1 C terminal and other components of exocyst complex	Blocks endocytic recycling	Block vesicle tethering and fusion with plasma membranes	40 µM in plants. 100 μM (§)	None	(Zhang et al., 2016), (Fujimoto et al., 2019)
*NIH-12848*	PI5P4Kγ	Allosteric non-ATP-competitive inhibitor	Reduces Notch recycling	Not reported	10–30 µM (§)		Clarke et al. (2015), Zheng and Conner (2018b)
	3.3 μM (*)						
*Apilimod*	PIKfyve	ATP-competitive inhibitor	Inhibits recycling of B1-integrin. Also affects the lysosomal pathway	Alters membrane integrity and morphology. Cause vacuolization	1 µM (§). 60 mg/kg (#)	Under evaluation for COVID-19 and Lymphoma treatment	Gayle et al. (2017), Giridharan et al. (2022)
	14 nM (*)						
*MLi2*	LRRK2	ATP-competitive inhibitor	Induces rapid recycling of RAB10 vesicles	Not reported	1 µM (§). 10 mg/kg (#)	None	Fell et al. (2015), Lobbestael et al. (2016), Liu et al. (2020), Mamais et al. (2021)
	0.76 nM (*)						

(*): IC_50_ in recombinant protein assay.

(§): in mammalian cells.

(#): in rodents.

### Lysosomotropic inhibitors

Many compounds belonging to the family of antimalarial agents, such as chloroquine and primaquine, were introduced as recycling inhibitors (van Weert et al., 1995; van Weert et al., 2000). These small molecules are weak bases, that once protonated, accumulate in endosomes causing both neutralization and deacidification of endosomal pH, thus resulting in endosomal recycling inhibition.

Primaquine and chloroquine are antiviral and antiparasite compounds used to block Zika, Ebola and malaria-related infections (Naghipour et al., 2020; Persoons et al., 2021). Moreover, primaquine and Chloroquine were recently under evaluation for therapeutic treatment of autoimmune diseases (Rainsford et al., 2015). As generic medications, huge efforts have been made to repurpose Chloroquine and Primaquine (and all others lysosomotropic drug) for cancer treatment. However, results coming from these studies are puzzling, thus limiting the employment of lysosomotropic drugs in both basic and clinical research (Ashley et al., 2014). Similar consideration must be made for BafilomycinA1, a macrolide antibiotic that blocks receptor recycling by inhibiting V-ATPase proton pump (Yoshimori et al., 1991). The V-ATPase consists of two main multisubunit complexes named V0 and V1. Structural studies and biochemical experiments defined that BafilomycinA1 causes steric hindrance between elements of V0 subunit, resulting in inhibition of V-ATPase activity (Wang et al., 2021).

### Exocyst complex inhibitor

The Exocyst complex mediates the tethering of secretory vesicles to plasma membrane, a trafficking step that anticipate the SNARE-mediated vesicle fusion (Mei and Guo, 2018). This multisubunit complex is composed by Sec3, Sec5, Sec6, Sec8, Exo84, and Exo70 (Martin-Urdiroz et al., 2016). Endosidin2, an exocyst complex inhibitor, interacts with Exo70 causing reduction of transferrin recycling in mammalian cells (Zhang et al., 2016).

### Kinase inhibitors

LRRK2 is a serine/threonine-protein kinase activated by oxidative, endolysosomal and autophagic stressors. LRRK2 phosphorylates a broad range of endocytic recycling regulators including the small GTPase Rab8A and Rab10 (Steger et al., 2017; Dhekne et al., 2018; Bonet-Ponce et al., 2020; Herbst et al., 2020; Kuwahara et al., 2020). Selective inhibition of LRRK2 by MLi2, an ATP-competitive inhibitor induces rapid recycling of RAB10 vesicles, thus reducing endocytic defects caused by hyperactivation of LRRK2 signalling in a murine model of Parkinson disease (Scott et al., 2017).

In contrast to the protein kinases, members of the lipid kinase family have less structural similarity in enzyme’s active site, simplifying generation of isoform-selective inhibitors (Roskoski, 2016). As an example, reduction of 5′ phosphorylated phosphoinositide signalling can be obtained using both competitive and non-competitive ATP inhibitors. Apilimod, an ATP competitive inhibitor, and NIH-12848, a non-competitive ATP inhibitor, reduce endocytic recycling of internalized cargoes by blocking the activity of PIKfyve and PI5P4Kγ lipid kinases, respectively (Clarke et al., 2015; Sbrissa et al., 2018; Baranov et al., 2020). Despite these findings, most studies are currently focused on the role of both Apilimod and NIH-12848 in endolysosomal regulation and its impact in immune-regulatory associated diseases (Gayle et al., 2017; Poli et al., 2021). Whether the endocytic recycling and the lysosomal trafficking pathways are linked by the 5′ phosphorylated phosphoinositide signalling is still unclear.

### Small GTPase inhibitors

Small GTPases are enzymes that catalyse the hydrolysis of guanosine triphosphate (GTP) and the subsequent generation of guanosine diphosphate (GDP). Activation of small GTPase is controlled by guanine nucleotide exchange factors (GEFs) which promote the release of GDP and the subseqeunt GTP loading. Conversely, GTPase-activating proteins (GAPs) bind to activated small GTPase to stimulate their GTPase activity. Small GTPase targeting might be used to control main steps involved in membrane trafficking including vesicle generation, transport, and fusion. Historically, the evidence of small GTPase targeting potential in membrane transport was demonstrated by Brefeldin A (BFA), a fungal toxin able to block ARF small GTPase activity (Helms and Rothman, 1992; Reiner and Lundquist, 2018). BFA is a protein-protein interaction inhibitor that disrupts the interaction between Arf1 and GBF1, an Arf GEF (Rouhana et al., 2013). Optimization of this protein-protein inhibitor has led to the development of LG186. LG186 is a selective Arf1-GBF1 interaction inhibitor, used to block, in addition to common Golgi-localized ARF1 trafficking pathways, CLIC/GEEC internalization (Klausner et al., 1992; Boal et al., 2010; Sathe et al., 2018).

However, the targeting of small GTPase by small molecule inhibitors remains difficult (Gray et al., 2020). At this regard, novel approaches, that use synthetic peptides and covalent small molecules, are now employed to prevent the interaction between small GTPase and their effector proteins and hence small GTPase signalling (Ali et al., 2019). As an example, the stapled peptide RFP14 blocks the interaction between Rab25 and FIP2, a Rab25 effector protein, causing a decrease of RAB25-driven cell proliferation (Mitra et al., 2017). A similar approach was employed to achieve RAB27 pathway inhibition, a key player in exosome secretion. Nexinhib20, a small molecule targeting the interaction between Rab27a and its effector JFC1, was found to regulate exocytosis-dependent neutrophil’s function and exosome secretion both *in vitro* and *in vivo* models of inflammation, a context in which exosome secretion and recycling are pivotal pathways for cancer disease progression (Johnson et al., 2016). Lastly, by taking advantage of two residues, that are unique to Rab27A and Rab27B, among the over 60 Rab family proteins (i.e., C123 and C188), a recent report identifies two covalent ligands (A01 and B01) that react preferentially with these cysteines, paving the way for future development of covalent RAB27A signalling inhibitors (Jamshidiha et al., 2022).

## Conclusion

The pharmacological targeting of both endocytosis and endosomal recycling pathways has the potential to improve therapeutic treatment of Mendelian genetic diseases, parasites/virus infections, neurodegeneration and cancers. However, due to the pleiotropic behaviour of endocytic genes, it remains difficult to hijack specific transport itineraries, as observed for Endosidin 2, a Exo70 small molecule inhibitor, that affects both constitutive exocytosis and receptor recycling. In parallel, drug repurposing has been explored to evaluate the therapeutic potential of membrane trafficking targeting in human diseases. For instance, Chlorpromazine, a GPCR antagonist used as an antipsychotic medication, was found to block dynamins activity and hence endocytosis of surface proteins. Despite these considerations, the druggability of many trafficking molecules is still unexplored.

In recent years, the development of both virtual screenings and cell-based functional assays have been employed for identification of novel trafficking inhibitors with improved target specificity and pathways selectivity. In this context, synthetic peptides, or covalent inhibitors able to block Rab small GTPases function could address selectivity issues in membrane trafficking inhibition.
