# Corrigendum: Small molecules targeting endocytic uptake and recycling pathways

**DOI:** 10.3389/fcell.2023.1274467

**Published:** 2023-08-18

**Authors:** Giampaolo Placidi, Clara Mattu, Gianluca Ciardelli, Carlo Cosimo Campa

**Affiliations:** ^1^ Italian Institute for Genomic Medicine, Candiolo, Italy; ^2^ Department of Mechanical and Aerospace Engineering, Politecnico di Torino, Turin, Italy; ^3^ Chemical-Physical Processes, National Research Council (CNR-IPCF), Pisa, Italy; ^4^ Candiolo Cancer Institute, FPO-IRCCS, Candiolo, Italy

**Keywords:** endocytosis, endocytic recycling, small molecules, inhibitors, mechanism of action

In the published article, there was an error in **Affiliation 4**. Instead of “Candiolo Cancer Institute, Candiolo, Italy”, it should be “Candiolo Cancer Institute, FPO-IRCCS, Candiolo, Italy”.

The authors apologize for this error and state that this does not change the scientific conclusions of the article in any way. The original article has been updated.

