# Supplementary material for: Small molecules targeting endocytic uptake and recycling pathways
Source: Front Cell Dev Biol. 2023 Mar 10;11:1125801. doi: 10.3389/fcell.2023.1125801 (PMC10036367; doi:10.3389/fcell.2023.1125801)
Supplement: Supplementary file 1 [file DataSheet1.PDF]

SUPPLEMENTARY FIGURE 1

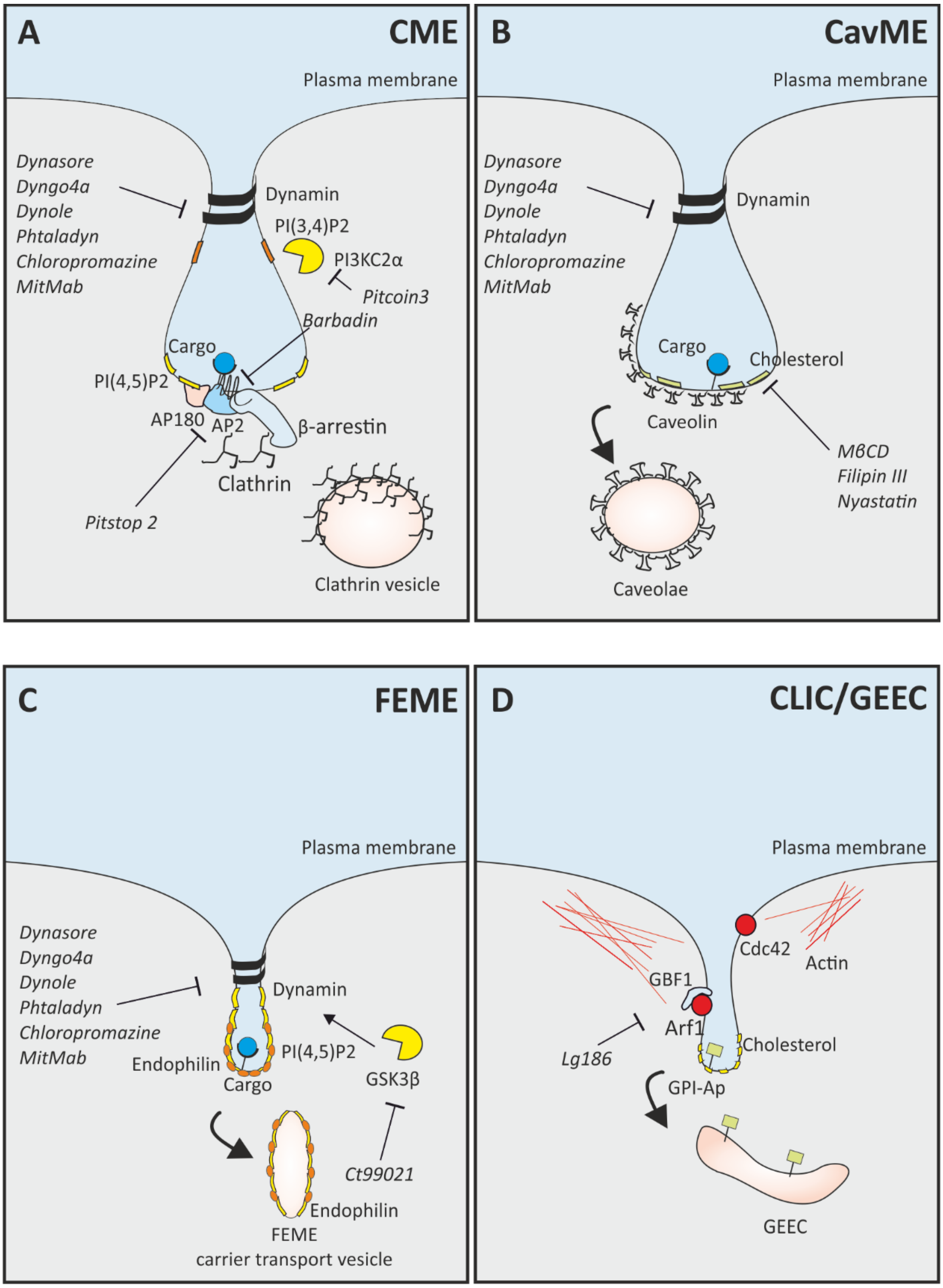

SUPPLEMENTARY FIGURE 1. Snapshots on main pathways controlling internalization of cell surface receptors:

A) Schematic representation of clathrin-mediated endocytosis (CME) inhibitors. After ligand binding, surface GPCR is internalized through recruitment of both AP2 and β-arrestin, two CLASPs proteins, thus guiding clathrin association to the new-born vesicle. Dynamin enzymes regulate pinch-off of the clathrin-coated vesicle from the cell surface. Available inhibitors for both AP2/β-arrestin and clathrin/CLASPs interactions as well as dynamin activity are listed.

B) Schematic representation of Caveolae mediated endocytosis (CavME) inhibitors. Caveolae are structures enriched of both lipids and cholesterols. Caveolin proteins induce membrane curvature and coat assembly to the growing vesicle. Available inhibitors for cholesterol-enriched domain and dynamin activity are listed.

C) Schematic representation of fast endophilin-mediated endocytosis (FEME) inhibitors. The rapid dephosphorylation of ligand-induced PI(3,4,5)P3 back into PI(4,5)P2 on cell membrane promotes recruitment of Endophilin. By binding to protein cargoes and phosphoinositides, endophilin induces membrane curvature and membrane scission in co-operation with dynamin, a GSK3β substrate, and actin. Available inhibitors for: dynamin and GSK3β are listed.

D) Schematic representation of clathrin-independent carrier/glycosylphosphatidylinositol-anchored protein enriched early endocytic compartment endocytosis (CLIC/GEEC) inhibitors. Internalization of Glycosylphosphatidylinositol-anchored proteins (GPI-Aps are abundant in lipid rafts. Arf1 and Cdc42 small GTPases promote actin polymerization and subsequent membrane curvature and vesicle fission. Available inhibitor for Arf1 is listed.

SUPPLEMENTARY FIGURE 2

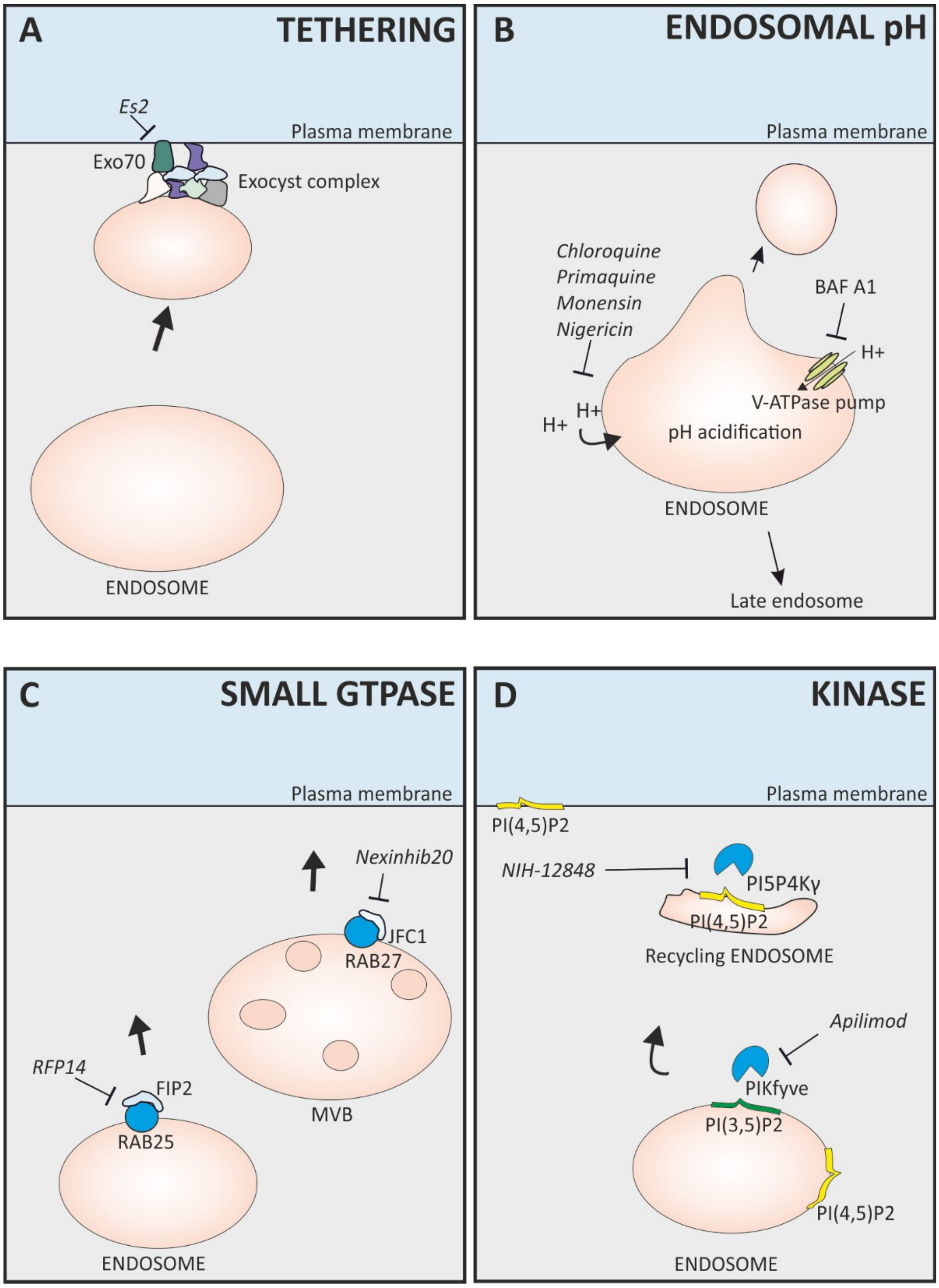

SUPPLEMENTARY FIGURE 2. Snapshots on main pathways controlling delivery of receptors to cell surface:

A) Schematic representation of tethering inhibitors. Components of the exocyst complex mediate the fusion of recycling vesicles with plasma membrane. Available inhibitor for Exo70 subunit is listed.

B) Schematic representation of endosomal pH inhibitors. Maturation of the endocytic membranes require pH acidification. Accumulation of lysosomotropic (weak bases positively charge) drugs in endosome prevent endosomal acidification. In parallel, V-ATPase activity reduce endosomal pH. Available inhibitor for endosomal pH are listed.

C) Schematic representation of small GTPase inhibitors. Interaction between active Rab25 and Rab27 small GTPases FIP2 and JFC1 effectors control exocytosis from endosome and multivesicular bodies. Available protein-protein interaction inhibitors for these molecular switches are listed.

D) Schematic representation of kinase inhibitors. Signalling lipids are main regulators of trafficking events. Generation of PI(5)P-derivates including PI(3,5)P2 and are fundamental for endocytic recycling pathway. PI(3,5)P2 is produced by PIKfyve, is generated by several enzymes including PI5P4Kγ. However the source of endocytic PI(4,5)P2 is debated. Available lipid kinase inhibitors affecting endocytic recycling are listed.
